# Prevalence of malnutrition and appropriateness of nutritional support in hospitalized patients: a GLIM-based study

**DOI:** 10.3389/fnut.2025.1667821

**Published:** 2025-10-10

**Authors:** Li-Min Deng, Shi-Ping Liu, Jian-Feng Long, Hui Mo, Min Liu, Ju-Ying Liu, Yun-Lin Zhang, Da-Feng Xu, Hui-Zhi Yu, Li Chen, Hua Cai, Shui-Bing Yang, Min Xiang, Min Liu, Yan Sun, Zhi-Juan Xie, Min-Ming Zeng, Guo-Xin Xie

**Affiliations:** ^1^Department of Clinical Nutrition, The Second Xiangya Hospital of Central South University, Changsha, Hunan, China; ^2^Department of Nutrition, The First People’s Hospital of Changde City, Changde, Hunan, China; ^3^Department of Clinical Nutrition, The Third Xiangya Hospital of Central South University, Changsha, Hunan, China; ^4^Department of Clinical Nutrition, Xiangya Hospital of Central South University, Changsha, Hunan, China; ^5^Department of Nutrition, The Second People’s Hospital of Xiangxiang City, Xiangtan, Hunan, China; ^6^Department of Nutrition, The First People’s Hospital of Chenzhou City, Chenzhou, Hunan, China; ^7^Department of Nutrition, The People’s Hospital of Yueyang City, Yueyang, Hunan, China; ^8^Department of Nutrition, Zhuzhou Central Hospital, Zhuzhou, Hunan, China; ^9^Department of Nutrition, The People’s Hospital of Hunan Province, Changsha, Hunan, China; ^10^Department of Nutrition, The First People’s Hospital of Huaihua City, Huaihua, Hunan, China; ^11^Department of Nutrition, The First People’s Hospital of Henyang City, Henyang, Hunan, China; ^12^Department of Nutrition, Shaoyang Central Hospital, Shaoyang, Hunan, China; ^13^Department of Nutrition, Changsha Central Hospital of University of South China, Changsha, Hunan, China; ^14^Department of Nutrition, The First Affiliated Hospital of University of South China, Henyang, Hunan, China; ^15^Department of Nutrition, The First Affiliated Hospital of Shaoyang University, Shaoyang, Hunan, China; ^16^Department of Nutrition, Xiangtan Central Hospital, Xiangtan, Hunan, China

**Keywords:** hospitalized patients, nutritional risk, malnutrition, nutritional therapy, GLIM criteria

## Abstract

**Background:**

To investigate the current status of nutritional risk, malnutrition, and nutritional therapy among hospitalized patients in Hunan Province, in order to provide evidence for optimizing clinical nutrition decision-making.

**Methods:**

A multi-stage continuous sampling method was applied to survey hospitalized patients across 16 hospitals in Hunan Province from March 2022 to August 2023. Nutritional risk was assessed using the Nutritional Risk Screening 2002 (NRS2002), while malnutrition was diagnosed according to the Global Leadership Initiative on Malnutrition (GLIM) criteria. Nutritional intervention status during hospitalization was recorded.

**Results:**

Among 3,189 participants, 36.4% (1,160/3,189) were identified as being at nutritional risk (NRS2002 ≥ 3), and of these, 68.2% (791/1,160) were diagnosed with malnutrition. Malnourished patients were older (65 ± 15 vs. 60 ± 13 years, *p* < 0.01), had lower BMI (20.2 ± 3.9 vs. 23.8 ± 3.5, p < 0.01), and incurred higher hospitalization costs (¥12,448.3 ± 12,064.3 vs. ¥10,070.3 ± 12,568.4, *p* = 0.038). High prevalence of nutritional risk and malnutrition was observed in infectious diseases, oncology, hematologic disorders, and respiratory diseases (prevalence>40%). Although 88.5% of malnourished patients received nutritional interventions, 10.9% received no support. Furthermore, 51.2% of patients without nutritional risk received unnecessary nutritional interventions.

**Conclusion:**

This study underscores a dual challenge in clinical nutritional management: insufficient support for high-risk patients alongside the overuse of nutritional therapy in low-risk populations. There is an urgent need to implement standardized protocols for screening, assessment, diagnosis, intervention, and monitoring to improve clinical outcomes and promote the rational allocation of healthcare resources.

**Clinical trial registration:**

https://www.clinicaltrials.gov/study/NCT05694104? term=NCT05694104&rank=1, Identifier NCT05694104.

## Introduction

Nutritional risk is defined as the existing or potential likelihood of adverse clinical outcomes associated with nutrition-related factors. The Nutrition Risk Screening 2002 (NRS 2002), recommended by the European Society for Clinical Nutrition and Metabolism (ESPEN), is the first evidence-based nutritional assessment and screening tool developed for hospitalized patients (1, 2). In 2018, the Global Leadership Initiative on Malnutrition (GLIM), established by four major international nutrition societies, introduced standardized criteria for malnutrition assessment, which were subsequently updated in April 2025 (3, 4). The GLIM criteria represent a major advancement in malnutrition diagnosis by offering a standardized, evidence-based, and globally applicable framework. Their use improves the accuracy of diagnosis, enhances the prediction of clinical outcomes, and facilitates targeted interventions. In this study, NRS 2002 was employed to screen the nutritional risk of inpatients across 16 hospitals in Hunan Province in China. Patients identified at risk were further assessed using the GLIM criteria. Additionally, the current status of nutritional support was examined and analyzed. The findings aim to provide a reference for optimizing clinical nutritional decision-making in hospitalized patients.

A consecutive fixed-site sampling approach was adopted to survey newly admitted inpatients at 16 hospitals across Hunan Province, including Xiangya Hospital of Central South University, the Second Xiangya Hospital of Central South University, Hunan Provincial People’s Hospital, and the First People’s Hospital of Changde City et al., from March 2022 to August 2023. This study was conducted as part of the National Nutrition Data Survey project overseen by the National Health Commission.

*Inclusion criteria*: (1) patients aged 18–90 years old; (2) hospitalization within 24–48 h, non-emergency patients; (3) provision of informed consent.

*Exclusion criteria*: (1) inpatients with mental illnesses or memory disorders who were unabe to answer the questions correctly; (2) critically ill inpaients and those who lacked behavioral abilities.

## Methods

A total of 16 secondary and tertiary hospitals were selected for the study. At each site, researchers employed a continuous convenience sampling approach from the initiation date until a predetermined sample size was achieved (total *N =* 200, with at least 25 cases per systemic disease category). Trained nutritionists or clinicians conducted structured face-to-face interviews with each participant using a validated case report form (CRF). Data collected encompassed patient demographics (gender, age, ethnicity, education level), admission and discharge dates, emergency admission status, primary diagnosis, anthropometric measures (height, weight, body mass index, waist and hip circumferences), laboratory test results, body composition analyses, NRS 2002 screening scores, GLIM-based nutritional diagnoses, types of in-hospital nutritional interventions, clinical outcomes at discharge, and total hospitalization costs. Nutritional interventions comprised standard hospital diet, oral nutritional supplements (ONS), enteral nutrition via tube feeding, and parenteral nutrition. The latter involved intravenous administration of amino acids, fat emulsions, dextrose, or total nutrient admixtures (TNA).

### Statistics

Statistical analyses were performed using SPSS software (version 27.0). Categorical variables were summarized as frequencies and percentages, and group comparisons were made using the chi-square (*χ*^2^) test. Continuous variables were expressed as mean ± standard deviation (SD), and intergroup differences were assessed using the Student’s *t-*test. A *p*-value below 0.05 was considered statistically significant.

## Results

### Characteristics of the study population

A total of 3,189 hospitalized patients from across Hunan Province were included in this study. Among them, 1,160 patients (36.4%) were identified as being at nutritional risk, defined by an NRS-2002 score ≥3. According to the GLIM criteria, which require the presence of nutritional risk (NRS-2002 score ≥3) plus at least one phenotypic and one etiologic criterion, 791 patients (24.8%) were diagnosed with malnutrition. The study population had a mean age of 61 years, with 1863 participants (58.4%) being male and 1,326 (41.6%) female. Compared to non-malnourished patients, those in the malnourished group were older, had a lower body mass index (BMI), and showed significantly reduced hemoglobin and albumin levels. Additionally, total hospitalization costs were higher among malnourished patients. Nutritional interventions were provided to 88.5% of malnourished patients, while 10.9% received no form of nutritional support. Among non-malnourished patients, 51.2% also received nutritional interventions. Detailed results are presented in Table 1.

**Table 1:** Baseline characteristics of hospitalized patients in Hunan province.

Variable	Total (*N =* 3,189)	Malnutrition (*N =* 791)	Non-malnutrition (*N =* 2,398)	*P*
Nutritional risks				
Yes (≥3)	1,160 (36.4%)	791 (100%)	369 (15.4%)	<0.01
No (<3)	2029 (63.6%)	0 (0%)	2029 (84.6%)	<0.01
Genders				
Male	1863 (58.4%)	464 (58.7%)	1,399 (58.3%)	0.901
Female	1,326 (41.6%)	327 (41.3%)	999 (41.7%)	
Age	61 ± 14	65 ± 15	60 ± 13	<0.01
Height	161.2 ± 7.8	160.9 ± 8.2	162.8 ± 7.5	0.007
Weight	59.5 ± 12.7	52.2 ± 11.1	63.1 ± 11.5	<0.01
BMI	22.5 ± 4.1	20.2 ± 3.9	23.8 ± 3.5	<0.01
Waist circumference (cm)	84.9 ± 11.0	77.0 ± 11.9	86.1 ± 10.0	<0.01
Hip circumference (cm)	90.2 ± 9.2	86.5 ± 8.7	92.8 ± 8.4	<0.01
Laboratory tests				
White blood cell count (×10^9^L)	7.1 ± 4.9	7.2 ± 3.5	7.0 ± 5.1	0.711
Hemoglobin	121 ± 25	112 ± 26	127 ± 23	<0.01
Blood platelet count (×10^9^L)	213 ± 89	225 ± 112	210 ± 72	0.162
Fasting blood sugar	7.0 ± 3.9	6.5 ± 3.4	7.6 ± 4.9	0.002
Total cholesterol	4.6 ± 1.9	4.3 ± 1.2	4.7 ± 2.4	0.018
Low-density lipoprotein Cholesterol	2.6 ± 2.6	2.4 ± 0.9	2.7 ± 1.3	0.006
Triglycerides	2.1 ± 3.7	1.4 ± 1.2	2.3 ± 3.8	0.01
Creatinine	93.5 ± 122.1	95.8 ± 130	92.8 ± 111.2	<0.01
Albumin	38.6 ± 5.9	36.3 ± 5.8	39.9 ± 5.5	<0.01
Whether nutritional intervention during hospitalization				
Yes	1928 (60.5%)	700 (88.5%)	1,228 (51.2%)	<0.01
No	1,221 (38.3%)	86 (10.9%)	1,135 (47.3%)	
Unknown	40 (1.3%)	5 (0.6%)	35 (1.5%)	
Death				
Yes	11 (0.3%)	4 (0.5%)	7 (0.3%)	0.148
No	3,177 (99.6%)	786 (99.4%)	2,391 (99.7)	
Unknown	1	1 (0.1%)	0	
Hospital fees	11254.3 ± 12362.3	12448.3 ± 12064.3	10070.3 ± 12568.4	0.038

BMI, Body Mass Index.

### Nutritional risk screening results

Among the patients surveyed in Hunan Province, 1,160 were identified as being at nutritional risk (NRS-2002 score≥3). The study population was stratified by primary disease diagnosis. Compared with patients not at nutritional risk, those identified as at risk showed significantly higher proportions of infectious and contagious diseases, malignancies, hematologic and hematopoietic disorders, respiratory diseases, digestive system diseases, musculoskeletal disorders, immune system diseases, and malnutrition-related conditions (all *p* < 0.05). Conversely, a significantly lower proportion of at-risk patients were diagnosed with endocrine, nutritional and metabolic disorders, as well as circulatory system diseases (all p < 0.05), as detailed in Table 2.

**Table 2:** Nutritional risk identified by NRS-2002 among patients with different disease diagnoses.

Types of disease diagnoses	Nutritional risk (*N =* 1,160)	No nutritional risk (*N =* 2029)	Total (*N =* 3,189)	*χ* ^2^	*P*
Communicable and infectious diseases					
Yes	25 (2.2%)	3 (0.1%)	28 (0.9%)	34.170	<0.001
No	1,135 (97.8%)	2026 (99.9%)	3,161 (99.1%)		
Tumors					
Yes	298 (25.7%)	318 (15.7%)	616 (19.3%)	47.516	<0.001
No	862 (74.3%)	1711 (84.3%)	2,573 (80.7%)		
Blood and hematopoietic organ diseases					
Yes	36 (3.1%)	7 (0.3%)	43 (1.3%)	42.218	<0.001
No	1,124 (96.9%)	2022 (99.7%)	3,146 (98.7%)		
Endocrine, nutritional and metabolic diseases					
Yes	211 (18.2%)	588 (29.0%)	799 (25.1%)	45.762	<0.001
No	949 (81.8%)	1,441 (71.0%)	2,390 (74.9%)		
Nervous system disease					
Yes	179 (15.4%)	322 (15.9%)	501 (15.7%)	0.107	0.743
No	981 (84.6%)	1707 (84.1%)	2,688 (84.3%)		
Diseases of the eyes, ears, nose, throat and other accessory organs					
Yes	12 (1.0%)	25 (1.2%)	37 (1.2%)	0.251	0.616
No	1,148 (99.0%)	2004 (98.8%)	3,152 (98.8%)		
Circulatory system diseases					
Yes	244 (21.0%)	588 (29.0%)	832 (26.1%)	24.162	<0.001
No	916 (79.0%)	1,441 (71.0%)	2,357 (73.9%)		
Respiratory diseases					
Yes	284 (24.5%)	332 (16.4%)	616 (19.3%)	31.224	<0.001
No	876 (75.5%)	1,697 (83.6%)	2,573 (80.7%)		
Digestive diseases					
Yes	323 (27.8%)	373 (18.4%)	696 (21.8%)	38.723	<0.001
No	837 (72.2%)	1783 (81.6%)	2,493 (78.2%)		
Diseases of the skin and subcutaneous tissues					
Yes	5 (0.4%)	7 (0.3%)	10 (0.4%)	0.007^*^	0.935^*^^*^
No	1,155 (99.6%)	2022 (99.7%)	2,798 (99.6%)		
Musculoskeletal disorder					
Yes	22 (1.9%)	18 (0.9%)	40 (1.3%)	6.072	0.014
No	1,138 (98.1%)	2011 (99.1%)	3,149 (98.7%)		
Immune system diseases					
Yes	11 (0.9%)	2 (0.1%)	13 (0.4%)	11.116^*^	0.001^*^^*^
No	1,149 (99.1%)	2027 (99.9%)	3,176 (99.6%)		
Diseases of the genitourinary system					
Yes	184 (15.9%)	327 (16.1%)	511 (16.0%)	0.035	0.851
No	976 (84.1%)	1702 (83.9%)	2,678 (84.0%)		
Pregnancy, childbirth, and postnatal comorbidities					
Yes	2 (0.2%)	8 (0.4%)	10 (0.3%)	0.561^*^	0.454^*^^*^
No	1,158 (99.8%)	2021 (99.6%)	2,806 (99.7%)		
Congenital malformations/chromosomal abnormalities					
Yes	0 (0.0%)	0 (0.0%)	0 (0.0%)	/	/
No	1,160 (100.0%)	2029 (100.0%)	3,189 (100.0%)		
Malnutrition-related diseases					
Yes	12 (1.0%)	4 (0.2%)	16 (0.5%)	10.366	0.001
No	1,148 (99.0%)	2025 (99.8%)	3,173 (99.5%)		
Unknown					
Yes	0 (0.0%)	0 (0.0%)	0 (0.0%)	/	/
No	1,160 (100.0%)	2029 (100.0%)	3,189 (100.0%)		

*Yates’s correction for continuity; **Fisher’s exact probability test, and the rest are Pearson’s chi-squared test.

**Figure 1:**
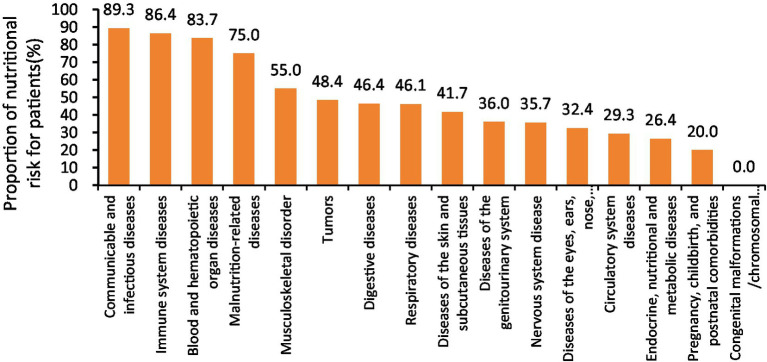
NRS 2002 nutritional risk among patients with different disease diagnosis types.

Furthermore, disease categories with a nutritional risk prevalence exceeding 40% included infectious and contagious diseases, immune system disorders, blood and hematopoietic organ diseases, malnutrition-related conditions, musculoskeletal disorders, malignancies, digestive diseases, respiratory diseases, and disorders of the skin and subcutaneous tissue, as illustrated in Figure 1.

### Malnutrition outcomes

According to the GLIM criteria for malnutrition diagnosis—which require the presence of nutritional risk (NRS-2002 score≥3), along with at least one phenotypic indicator and one etiologic indicator—a total of 791 patients (24.8%) in Hunan Province were diagnosed with malnutrition. The surveyed patients were stratified by primary disease diagnosis. Compared to patients without nutritional risk, those with nutritional risk were significantly more likely to be diagnosed with infectious and contagious diseases, malignancies, blood and hematopoietic organ diseases, respiratory diseases, digestive system diseases, immune system disorders, and malnutrition-related conditions (all *p* < 0.05). Conversely, lower proportions of nutritional risk were observed among patients with endocrine, nutritional and metabolic diseases, as well as circulatory system diseases (all p < 0.05), as shown in Table 3.

**Table 3:** Malnutrition of GLIM in patients with different disease diagnosis types.

cTypes of disease diagnoses	Malnutrition (*N =* 791)	Non-malnutrition (*N =* 2,398)	Total (*N =* 3,189)	*χ* ^2^	*P*
Communicable and infectious diseases					
Yes	23 (2.9%)	5 (0.2%)	28 (0.9%)	49.793	<0.001
No	768 (97.1%)	2,393 (99.8%)	3,161 (99.1%)		
Tumors					
Yes	246 (31.1%)	370 (15.4%)	616 (19.3%)	93.717	<0.001
No	545 (68.9%)	2028 (84.6%)	2,573 (80.7%)		
Blood and hematopoietic organ diseases					
Yes	31 (3.9%)	12 (0.5%)	43 (1.3%)	52.260	<0.001
No	760 (96.1%)	2,386 (99.5%)	3,146 (98.7%)		
Endocrine, nutritional and metabolic diseases					
Yes	127 (16.1%)	672 (28.0%)	799 (25.1%)	45.369	<0.001
No	664 (83.9%)	1726 (72.0%)	2,390 (74.9%)		
Nervous system disease					
Yes	97 (12.3%)	404 (16.8%)	501 (15.7%)	9.440	0.002
No	694 (87.7%)	1994 (83.2%)	2,688 (84.3%)		
Diseases of the eyes, ears, nose, throat and other accessory organs					
Yes	12 (1.5%)	25 (1.0%)	37 (1.2%)	1.168	0.280
No	779 (98.5%)	2,373 (99.0%)	3,152 (98.8%)		
Circulatory system diseases					
Yes	163 (20.6%)	669 (27.9%)	832 (26.1%)	16.399	<0.001
No	628 (79.4%)	1729 (72.1%)	2,357 (73.9%)		
Respiratory diseases					
Yes	223 (28.2%)	393 (16.4%)	616 (19.3%)	53.172	<0.001
No	568 (71.8%)	2005 (83.6%)	2,573 (80.7%)		
Digestive diseases					
Yes	239 (30.2%)	457 (19.1%)	696 (21.8%)	43.398	<0.001
No	552 (69.8%)	1941 (80.9%)	2,493 (78.2%)		
Diseases of the skin and subcutaneous tissues					
Yes	5 (0.6%)	7 (0.3%)	12 (0.4%)	1.041^*^	0.308
No	786 (99.4%)	2,391 (99.7%)	3,177 (99.6%)		
Musculoskeletal disorder					
Yes	13 (1.6%)	27 (1.1%)	40 (1.3%)	1.286	0.257
No	778 (98.4%)	2,371 (98.9%)	3,149 (98.7%)		
Immune system diseases					
Yes	10 (1.3%)	3 (0.1%)	13 (0.4%)	16.308^*^	<0.001
No	781 (98.7%)	2,395 (99.9%)	3,176 (99.6%)		
Diseases of the genitourinary system					
Yes	118 (14.9%)	393 (16.4%)	511 (16.0%)	0.956	0.328
No	673 (85.1%)	2005 (83.6%)	2,678 (84.0%)		
Pregnancy, childbirth, and postnatal comorbidities					
Yes	2 (0.3%)	8 (0.3%)	10 (0.3%)	0.000^*^	1.000
No	789 (99.7%)	2,390 (99.7%)	3,179 (99.7%)		
Congenital malformations/chromosomal abnormalities					
Yes	0 (0.0%)	0 (0.0%)	0 (0.0%)	/	/
No	791 (100.0%)	2,389 (100.0%)	3,189 (100.0%)		
Malnutrition-related diseases					
Yes	11 (1.4%)	5 (0.2%)	16 (0.5%)	14.367^*^	<0.001
No	780 (98.6%)	2,393 (99.8%)	3,173 (99.5%)		
Unknown					
Yes	0 (0.0%)	0 (0.0%)	0 (0.0%)	/	/
No	791 (100.0%)	2,398 (100.0%)	3,189 (100.0%)		

*Yates’s correction for continuity; **Fisher’s exact probability test, and the rest are Pearson’s chi-squared test.

**Figure 2:**
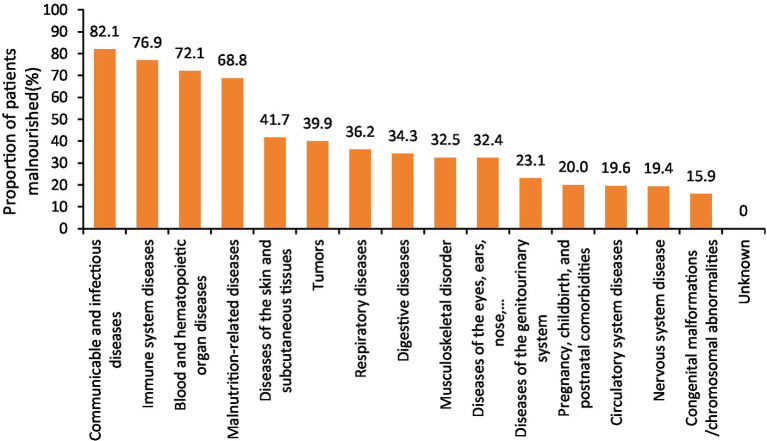
Malnutrition of GLIM in patients with different disease diagnosis types.

Furthermore, disease types with a malnutrition prevalence exceeding 30% included: infectious and contagious diseases, immune system disorders, blood and hematopoietic organ diseases, malnutrition-related conditions, skin and subcutaneous tissue disorders, tumors, respiratory diseases, digestive system diseases, musculoskeletal disorders, and diseases of the eyes, ears, nose, throat, and other accessory organs, as illustrated in Figure 2.

### Status of nutritional therapy

During hospitalization, a total of 1,928 patients (60.5%) received nutritional therapy interventions, while 1,221 patients (38.2%) did not receive any nutritional intervention, and the intervention status was unknown for 40 patients. Among those identified as being at nutritional risk, 83.3% received nutritional therapy, while 16.7% did not. Additionally, 48.5% of patients without nutritional risk received nutritional interventions. Among patients diagnosed with malnutrition according to the GLIM criteria, 89.1% received nutritional therapy, whereas 10.9% did not receive any intervention. By contrast, 51.2% of non-malnourished patients received nutritional support. Detailed data are presented in Table 4.

**Table 4:** Nutritional interventions for patients with different types of disease diagnosis.

Types of disease diagnoses	Nutritional interventions (*N =* 1928)	No nutritional interventions (*N =* 1,221)	Total (*N =* 3,149)	*χ* ^2^	*P*
Communicable and infectious diseases					
Yes	28 (1.5%)	0 (0%)	28 (0.9%)	17.891	<0.001
No	1900 (98.5%)	1,221 (100%)	3,121 (99.1%)		
Tumors					
Yes	406 (21.1%)	193 (15.8%)	616 (19.0%)	13.384	<0.001
No	1,522 (78.9%)	2028 (84.2%)	2,573 (81.0%)		
Blood and hematopoietic organ diseases					
Yes	39 (2.0%)	3 (0.2%)	42 (1.3%)	17.941	<0.001
No	1889 (98.0%)	1,218 (39.2%)	3,107 (98.7%)		
Endocrine, nutritional and metabolic diseases					
Yes	523 (27.1%)	273 (22.4%)	796 (25.3%)	8.997	0.003
No	1,405 (72.9%)	948 (77.6%)	2,353 (74.7%)		
Nervous system disease					
Yes	266 (13.8%)	220 (18.0%)	486 (15.4%)	10.207	0.001
No	1,662 (86.2%)	1,001 (82.0%)	2,688 (84.6%)		
Diseases of the eyes, ears, nose, throat and other accessory organs					
Yes	29 (1.5%)	7 (0.6%)	36 (1.1%)	5.732	0.017
No	1899 (98.5%)	1,207 (99.4%)	3,113 (98.9%)		
Circulatory system diseases					
Yes	462 (24.0%)	357 (29.2%)	819 (26.0%)	10.812	0.001
No	1,466 (76.0%)	864 (70.8%)	2,330 (74.0%)		
Respiratory diseases					
Yes	402 (20.9%)	208 (17.0%)	610 (19.4%)	6.968	0.008
No	1,526 (79.1%)	1,013 (83.0%)	2,539 (80.6%)		
Digestive diseases					
Yes	496 (25.7%)	188 (15.4%)	684 (21.7%)	46.906	<0.001
No	1,432 (74.3%)	1,033 (84.6%)	2,465 (78.3%)		
Diseases of the skin and subcutaneous tissues					
Yes	9 (0.5%)	3 (0.2%)	12 (0.4%)	0.963^*^	0.327
No	1919 (99.5%)	1,218 (99.8%)	3,137 (99.6%)		
Musculoskeletal disorder					
Yes	27 (1.4%)	12 (1.0%)	39 (1.2%)	1.066	0.302
No	1901 (98.6%)	1,209 (99.0%)	3,110 (98.8%)		
Immune system diseases					
Yes	12 (0.6%)	1 (0.1%)	13 (0.4%)	5.312^**^	0.021
No	1916 (99.4%)	1,220 (99.9%)	3,136 (99.6%)		
Diseases of the genitourinary system					
Yes	317 (16.4%)	190 (15.6%)	507 (16.1%)	0.429	0.512
No	1,611 (83.6%)	1,031 (84.4%)	2,642 (83.9%)		
Pregnancy, childbirth, and postnatal comorbidities					
Yes	6 (0.3%)	4 (0.3%)	10 (0.3%)	0.006^*^	0.936
No	1922 (99.7%)	1,217 (99.7%)	3,139 (99.7%)		
Congenital malformations/ chromosomal abnormalities					
Yes	0 (0.0%)	0 (0.0%)	0 (0.0%)	/	/
No	1928 (100.0%)	1,221 (100.0%)	3,149 (100.0%)		
Malnutrition-related diseases					
Yes	12 (0.6%)	2 (0.2%)	14 (0.4%)	3.552^*^	0.059
No	1916 (99.4%)	1,219 (99.8%)	3,135 (99.6%)		
Unknown					
Yes	0 (0.0%)	0 (0.0%)	0 (0.0%)	/	/
No	1928 (100.0%)	1,221 (100.0%)	3,149 (100.0%)		

*Yates’s correction for continuity; **Fisher’s exact probability test, and the rest are Pearson’s chi-squared test.

**Figure 3:**
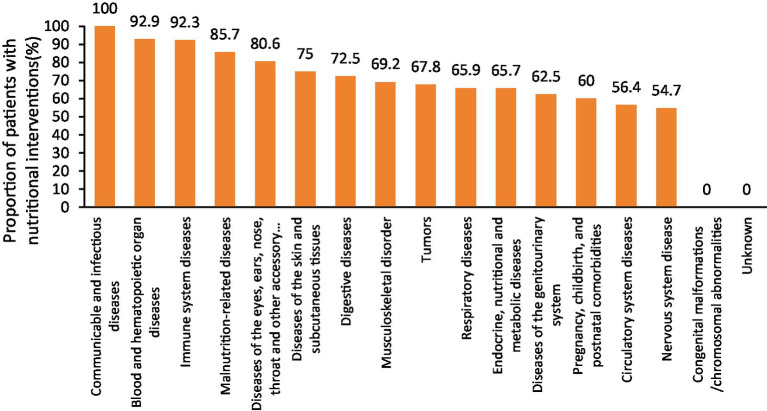
The proportion of nutritional intervention for patients with different disease diagnosis types.

A stratified analysis based on disease diagnosis type (excluding cases with unknown intervention status) revealed that, compared with patients who did not receive nutritional therapy, a significantly higher proportion of those who received nutritional interventions were diagnosed with infectious and contagious diseases, blood and hematopoietic organ diseases, immune system disorders, and diseases of the eyes, ears, nose, throat, and other accessory organs (all *p* < 0.05). In contrast, lower proportions of nutritional therapy were observed among patients with nervous system diseases and circulatory system diseases (both p < 0.05), as shown in Table 4. Moreover, disease categories with the highest rates of nutritional intervention (over 80%) included infectious and contagious diseases, blood and hematopoietic organ diseases, immune system diseases, malnutrition-related conditions, and diseases of the eyes, ears, nose, throat, and other accessory organs, as illustrated Figure 3.

## Conclusion

The NRS2002 is applicable for large-scale clinical nutritional risk screening. However, it has limitations in patients who are unconscious, have communication difficulties, cannot stand, or have edema/ascites, as these conditions impede accurate body mass measurement. In such cases, biochemical indicators should be used to guide appropriate nutritional therapy. This study found that 36.4% of hospitalized patients in Hunan Province were at nutritional risk. Stratified analysis by primary disease diagnosis showed that patients with infectious diseases, malignancies, hematologic and hematopoietic disorders, respiratory diseases, digestive system diseases, musculoskeletal conditions, immune-related diseases, and diseases related to malnutrition had significantly higher rates of nutritional risk and were more frequently diagnosed with malnutrition.

The GLIM criteria, as a standardized diagnostic framework, have been validated in prior studies to possess high sensitivity and specificity in hospital settings. They effectively identifying malnutrition prevalence and severity while aligning with other validated screens, making them suitable for standardized diagnosis and predicting clinical outcomes (5). Shi et al. (6) found that the GLIM criteria can identify malnutrition in elderly cancer patients and predict survival more accurately than traditional TNM classification for 1–2 year estimates. Yilmaz et al. (7) reported 82% of hematological malignancy patients were at risk via NRS-2002, with 25.8% diagnosed as malnourished by GLIM; this group had a 41.7% 1-year mortality rate, and malnutrition was an independent mortality risk factor regardless of age or disease duration. These findings support the use of GLIM as a predictive tool for patients’ outcome. In our study, 68.2% of patients identified as being at nutritional risk were diagnosed with malnutrition, including 68.8% of those with blood and hematopoietic organ diseases—a proportion higher than reported by Yilmaz et al.

This study revealed that 60.5% of hospitalized patients received nutritional therapy, while 38.2% did not. Among patients diagnosed with malnutrition, 88.5% received nutritional support—reflecting clinical recognition of its importance in this high-risk group. However, the substantial underutilization in the broader patient population underscores the need to improve systematic nutritional risk screening. Furthermore, 51.2% of patients without nutritional risk still received nutritional interventions, suggesting possible overtreatment and inefficient allocation of medical resources. These findings reveal inconsistencies in nutritional care practices within hospitals in Hunan Province and emphasize the urgency of implementing standardized, evidence-based clinical protocols.

In summary, over one-third of hospitalized patients were identified as being at nutritional risk, with the majority meeting the diagnostic criteria for malnutrition. Providing timely nutritional support to these high-risk patients can help reduce complications, accelerate recovery, and decrease hospitalization costs. Conversely, the provision of nutritional therapy to more than half of patients without nutritional risk indicates a substantial misallocation of healthcare resources. It is essential to strengthen nutritional care quality control and enhance professional training at both institutional and provincial levels. Clinical staff working in high-risk departments should adhere to standardized protocols covering screening, assessment, diagnosis, intervention, and monitoring. Implementing evidence-based and individualized nutritional care plans will improve patient outcomes and increase the efficiency of medical resource utilization.

## Data Availability

The raw data supporting the conclusions of this article will be made available by the authors, without undue reservation.
